# Optical method to preserve residual hearing in patients receiving a cochlear implant

**DOI:** 10.3389/fauot.2024.1376699

**Published:** 2024-04-15

**Authors:** Joaquin Cury, Arnaldo Rivera, Rebecca Schneider, Ray Tan, Xiaodong Tan, Claus-Peter Richter

**Affiliations:** 1 Department of Otolaryngology, Feinberg School of Medicine, Northwestern University, Chicago, IL, United States; 2 Department of Otolaryngology, School of Medicine, Missouri University, Columbia, MO, United States; 3 Hugh Knowles Center for Clinical and Basic Science in Hearing and Its Disorders, Northwestern University, Evanston, IL, United States; 4 Department of Biomedical Engineering, Northwestern University, Evanston, IL, United States; 5 Department of Communication Sciences and Disorders, Northwestern University, Evanston, IL, United States

**Keywords:** cochlear implants, hearing loss, cochlear implantation, hearing preservation, cochlear endoscopy

## Abstract

**Introduction:**

Worldwide, thousands of patients with severe to profound hearing loss restore their hearing with cochlear implant (CI) devices. Newer developments in electrode design and manufacturing and a better understanding of cochlear mechanics allow for conserving critical structures, often translating into serviceable residual hearing and improving device performance. Monitoring insertion speed and intraluminal pressure helps mitigate some of these challenges. However, the information becomes available after irreparable damage has occurred.

**Methods:**

We developed and tested a high-resolution optical system to navigate the intricate anatomy of the cochlea during electrode insertion. The miniaturized optical system was integrated in conventional cochlear implants electrode arrays and custom-made cochlear probes. Electrode insertion were conducted in eight cadaveric human temporal bones and video recordings were acquired. Micro-computed tomography (μCT) scans were performed to evaluate the position of the modified electrode arrays.

**Results:**

Full insertions of the modified CI electrode were successfully conducted and verified by μCT scans. Video recordings of the cochlear structures visible in scala tympani were acquired, and no scala migration was detected.

**Discussion:**

Surgeons can now follow the CI electrode’s path during its insertion and reduce cochlear damage through early interventions and steering of the CI electrode. Our device will be compatible with robotic platforms that are already available to insert these electrodes.

## 1 Introduction

Hearing loss is a global health crisis. According to the World Health Organization (WHO), over 1.5 billion people globally suffer from hearing loss, disabling 466 million of them (Olusanya et al., 2019; World Health Organization, 2021). The same reports suggest that the numbers will almost double by 2050 (Olusanya et al., 2019; World Health Organization, 2021). Unaddressed hearing loss costs the global economy approximately US$980 billion annually (World Health Organization, 2021). Furthermore, a recent meta-analysis suggested that hearing is a modifiable risk factor for dementia (Loughrey, 2022; Lin et al., 2023; Cantuaria et al., 2024) and that treating hearing loss will decrease the risk of long-term cognitive decline by 19% (Yeo et al., 2023). While mild and moderate hearing loss is treated with hearing aids, ~30 million severely to profoundly deaf patients could benefit from a cochlear implant (CI) to restore some of their hearing (World Health Organization, 2021). Of those who could benefit from a CI, as of 2024, ~1 million people have received a CI, with 60,000 additional individuals implanted annually (NIDCD, 2021).

While CIs are one of the most successful neural prosthetics, the surgery for electrode implantation is not without risks of further damaging the inner ear’s delicate structures and worsening hearing loss (Miranda et al., 2014; De Seta et al., 2017; Starovoyt et al., 2023). The inner ear is exposed during the CI surgery by drilling a narrow passage to the middle ear. The CI electrode is then typically placed into the scala tympani, a fluid-filled compartment of the inner ear. The insertion occurs through either the natural opening in the cochlea, the round window (RW), or a cochleostomy, an artificial inner ear opening. The CI electrode insertion reaches a critical point after 8–10 mm. At this location, the scala tympani abruptly changes its radius of curvature (1.6–2.6 mm) and turns toward the apex. Significant contact between the CI electrode and the cochlear wall occurs and can result in an abrupt increase in insertion force, leading to electrode buckling, potential tissue damage, and basilar membrane penetration (Eshraghi, 2006). Damage and possible misalignment of the CI electrode trigger inflammation, leading to subsequent loss of residual hearing in up to 32% of implantations (Hoskison et al., 2017). This percentage might even be greater, as suggested by studies in cadaveric human temporal bones, which have shown damage in up to 40% of the electrode insertions (Eshraghi et al., 2003; Mirsalehi et al., 2017). Therefore, preserving residual hearing is crucial as it can enhance the CI user’s performance and improve the recipient’s speech comprehension, music appreciation, and overall quality of life.

That efforts for atraumatic electrode insertion have surged is not surprising, and the CI industry, surgeons, and researchers have explored changes in the device and CI implantation surgery to maximize hearing preservation. The first debate was about the correct location for the opening for the CI electrode. Over time, two methods for making a cochleostomy have been established: the RW and an antero-inferior cochleostomy in the basal cochlear turn (Sikka et al., 2017; Avasarala et al., 2022). Several studies on the outcomes of electrode insertion on cochlear damage and hearing preservation during CI surgery did not identify a definitive advantage of the electrode insertion through a cochleostomy or RW (Havenith et al., 2013; Rajput and Nilakantan, 2019). Different from previous studies, Santa Maria et al. (2014) reported a benefit in hearing preservation using the cochleostomy method, whereas better hearing preservation was reported after using the RW approach (Causon et al., 2015; Avasarala et al., 2022).

Critical to the success of CI surgeries is also the cochlear electrode array, which is surgically inserted into the cochlea to stimulate the auditory nerve electrically. The precise placement of the electrode is essential for activating the spiral ganglion neurons, which are responsible for transmitting sound signals to the brain. To enhance hearing restoration outcomes, maintaining the structural integrity of the inner ear components during this insertion procedure is necessary. These components include the basilar membrane, which is important for sound frequency discrimination; the modiolus, the central core of the cochlea that contains the spiral ganglion neurons; and the cochlear wall, which preserves the cochlea’s internal environment.

Over the last two decades, efforts have been made to optimize the electrode array materials and their physical properties, including length, diameter, and compliance. The longest cochlear electrodes measure about 31 mm and are designed to be inserted as far as possible into a spiral structure with about 2.5 turns (Jagt et al., 2017).

Another approach to avoid cochlear damage during the surgery is monitoring the insertion process and taking preventative measures before the damage occurs. While various monitoring techniques exist to support the insertion process, such as impedance and insertion force measurements (Majdani et al., 2010; Miroir et al., 2012; Tan et al., 2013; Dong et al., 2021; Hafeez et al., 2021), cone-beam computed tomography (Bassiouni et al., 2014; Saeed et al., 2014), fluoroscopy (Perazzini et al., 2021), and electrocochleography recordings (O’Connell et al., 2017; Giardina et al., 2019), surgeons primarily rely on tactile feedback to establish the insertion trajectory and control the insertion process. This method can only detect increased resistance after the electrode contacts the cochlear wall, which potentially causes tissue trauma.

Innovative solutions have been explored, including robotic insertions, to achieve better control over the speed and force of electrode insertion into the cochlea (Kaufmann et al., 2020; Panara et al., 2021; De Seta et al., 2022), and optical techniques, such as optical coherence tomography (OCT) imaging, to determine the path and location of the advancing CI electrode (Starovoyt et al., 2022). Despite the potential benefits of OCT, the method faces certain constraints. Navigating the cochlear turns requires flexible waveguides with low propagation and bending losses. Without them, the effectiveness of OCT is limited to the initial few millimeters during the electrode insertion.

With the advent of nanocameras, new opportunities have arisen. Miniaturized cameras can provide real-time surgical field visualization, which is invaluable for surgeons when inserting CI electrodes. The further miniaturization of cameras and the progress in imaging technology seen in other fields (Kaur et al., 2020; Zhang et al., 2021; Kim et al., 2022; Duan et al., 2023; Lavenir et al., 2023) have enabled us to develop high-resolution endoscopic-like systems to navigate the intricate anatomy of the cochlea. Such a system not only assists in precise electrode insertion but also aids in identifying and avoiding potential obstacles, thereby reducing the risk of damage to delicate cochlear structures. These nanocamera systems could be further integrated with robotic platforms to enhance the procedure’s accuracy and safety. Moreover, this optical method can be used to evaluate the health of the cochlea, particularly the basilar membrane, before and after implantation; determine the optimal insertion depth and trajectory; and potentially foresee and avert postoperative complications.

In the present study, we describe an optical system that can be integrated with a cochlear electrode array, providing visual real-time monitoring of the cochlea inner structures during the insertion procedure (Cury and Richter, 2023).

## 2 Materials and methods

### 2.1 Optical system

The optical system incorporates a nanocamera, an illumination source, and an image postprocessing module. The acquired images are then sent to a computer for display. The nanocamera used in our study is the OVM6948 (Omnivision, Santa Clara, California, United States). The OVM6948 is currently the smallest commercially available camera in the world, with an integrated image array, signal processing, timing, and control circuitry all housed on a single integrated circuit. Its current dimensions are remarkably compact, 0.65 × 0.65 mm^2^, with a z height of 1.16 mm, and it offers a resolution of 200 × 200 pixels. The camera can capture high-quality images and video up to 30 frames per second. Despite its miniature size, the image sensor incorporates advanced imaging technology, including microlenses. Furthermore, the camera chip is designed to be power-efficient, boasting a low power consumption of just 25 mW.

To deliver light into the cochlea, we explored two micro-LED models emitting yellow light (λ = 591 nm), each with a different size. The first is model 0402 from Evemodel (China), and measures 1.0 × 0.5 × 0.4 mm^3^. The second model, the Nanopoint 0201 by SunLED, (California, USA), measures 0.65 × 0.35 × 0.20 mm^3^ and is currently the smallest micro-LED available on the market.

The module for postprocessing the images captured by the nanocamera OVM6948 comprises two main components: a video bridge chip, specifically the OV426 (Omnivision, California, USA), and a digital signal processor (DSP). The OV426 is mainly chosen for its compatibility with the OVM6948, offering integrated analog-to-digital conversion and a digital video parallel output. This chip converts the camera’s analog video signals into a digital format. After this initial processing, the DSP further processes the digital signals. The resulting video data, now fully processed, are transmitted to the computer via a USB connection. For the final step, the data are visualized using Amcap software, allowing for a detailed examination of the images captured by the nanocamera.

### 2.2 Integration of nanocameras in CI electrode arrays and development of cochlear probes

**FIGURE 1:**
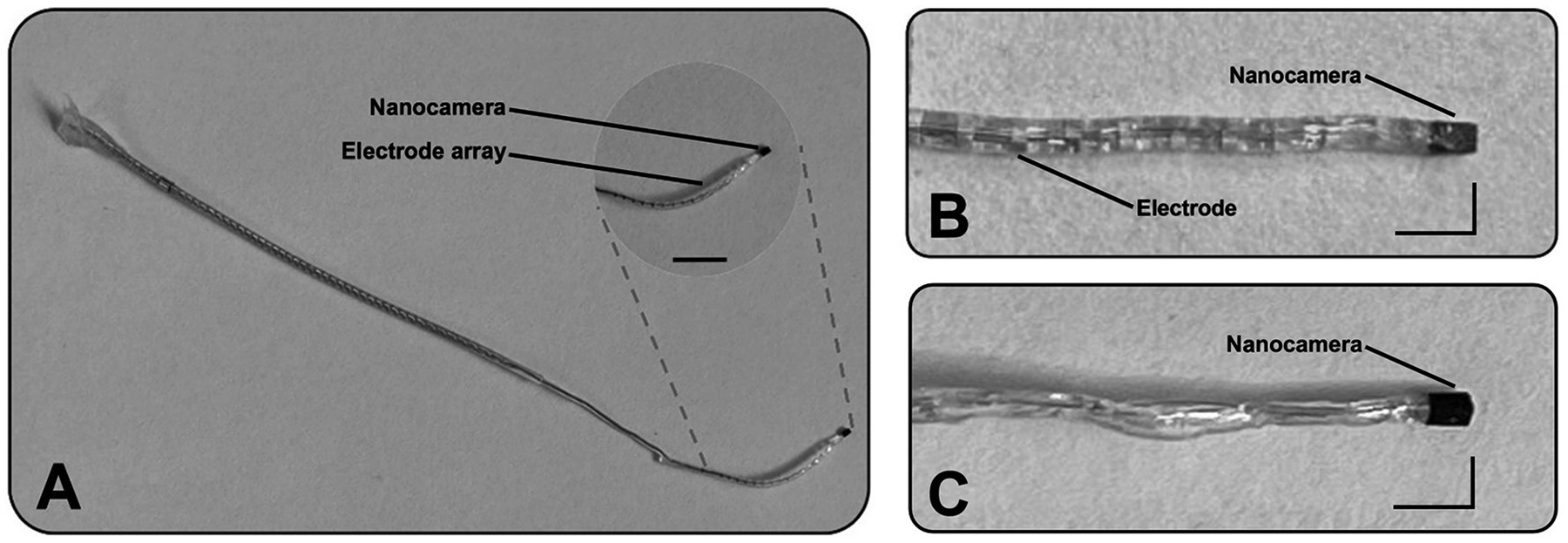
Illustration of two designs incorporating the optical system at their distal part. **(A)** The first prototype consists of a cochlear implant electrode array (J1–HiRes design from Advanced Bionics) with a nanocamera at its tip. The scale bar represents 5 mm. **(B)** Magnified view of the same prototype’s tip. **(C)** Magnified view of the second design. Scale L-bars: vertical and horizontal segments represent 1 and 2 mm, respectively.

This study modified CI electrode arrays (J1–HiRes design, Advanced Bionics, California, United States) and developed custom-made cochlear probes. The CI arrays and the custom probes were equipped with the OVM6948 nanocamera at their tips (Figure 1). To attach the camera to the tip of the electrode, we removed the three most distal ring electrodes of the CI arrays, reducing their length by ~3 mm. The wires previously used for these electrodes and the ground wire were repurposed to connect the nanocamera using conductive epoxy and further secured with ultraviolet (UV)–curable polymer OrmoComp^®^ (Kayaku Advanced Materials, Westborough, Massachussets, United States).

For the custom-made cochlear probes, specifically designed to capture video within the cochlea, we employed four silver-coated wires with an inner diameter of approximately 76 μm and a total diameter of approximately 140 μm (A–M Systems, USA) to establish the nanocamera connection. These wires were secured for consistency using the same conductive epoxy and UV-curable polymer. Once connected, the assembly was encapsulated in silicone to replicate the external structure of a conventional CI electrode, although without the ring electrodes (Figure 1C).

The light source for the nanocamera was strategically attached laterally to the optical system. To ensure the design’s compactness, the placement was such that the smaller dimension of the micro-LED contributed to a minimal increase in the overall width of the nanocamera’s assembly. To secure the micro-LEDs firmly and maintain the assembly’s integrity, we used UV-curable polymer OrmoComp^®^. Each prototype (modified CI electrode array and cochlear probe) utilized one version of micro-LED at a time rather than both versions simultaneously due to the limited space in the cochlea.

### 2.3 Testing of the nanocamera

To evaluate the performance of the optical system, we determined three parameters: spatial resolution, spatial frequency response, and optical distortion. To assess the spatial resolution, we captured an image on paper featuring a transition from black to white. We analyzed the intensity profile along a black-to-white transition using the ImageJ software. The camera system’s resolution was determined by a single value: the distance of the 10%−90% pixel value along a line along the black–white transition (Smith, 1997). The results, given in image pixel counts, were converted into micrometers using the nanocamera’s pixel size (1.75 μm; Omnivision, USA).

Using the edge response, we calculated the modulation transfer function (MTF; Smith, 1997) and quantified the system’s ability to preserve detail across various spatial frequencies. This process involved deriving the line spread function (LSF) from the edge response, followed by a Fourier transform on the LSF.

To analyze spherical distortions, we captured an image of a piece of 1-mm graph paper and compared it with the same image distortion-free as a reference. The distortion was quantified as the relative change of the distance from the image center to the distorted and corrected cross section of the graph paper lines close to the edge of the image taken (McReynolds and Blythe, 2005).

### 2.4 Cochlear samples and video recordings

In this study, we randomly selected eight cadaveric human temporal bones to evaluate the insertion of our modified CI electrodes and custom cochlear probes. We used human cadaveric temporal bones without known congenital or acquired malformations or a history of chronic otologic disease. The preparation of the temporal bone consisted of a cortical mastoidectomy with posterior tympanotomy, the exposure of the RW, and the removal of the RW overhang. After the RW niche was exposed, the RW membrane was removed with a right-angle pick. The electrode was inserted through the RW. The nanocamera confirmed visual placement as well as depth of insertion. Critical structures were identified and preserved through video feedback. The insertions were conducted by a neurotologist at the University of Missouri.

### 2.5 Micro-computed tomography imaging

Micro-computed tomography (μCT) scans were conducted to evaluate the position of the CI array, with the nanocamera attached, within the human cadaveric temporal bone. Each temporal bone was fixed, and the modified CI array was carefully inserted into the cochlea following a standard surgical procedure (Section 2.4). After the insertion, μCT scans were captured on a Siemens Inveon micro-positron emission tomography/computed tomography (PET/CT) system (Malvern, USA). The section of the temporal bone analyzed had dimensions of 66 × 66 × 52 mm^3^. From this section, 755 image slices were obtained, each with a separation of approximately 70 μm. The acquired data were reconstructed using the ImageJ software to generate two- and three-dimensional visualizations of the electrode’s placement with the nanocamera within the cochlea. The scans were analyzed to assess the positioning, depth, and potential damage caused by the electrode insertion.

### 2.6 Statement of ethics

The study protocol was in accordance with the ethical standards established in the 1964/2013 (7th revision) Declaration of Helsinki for research involving human subjects. Human cadavers were donated for teaching and research purposes. Human temporal bones were collected at the end of an anatomy class for medical students. Before their use in research, the temporal bones were irreversibly stripped of all identifiers, thus making it impossible to link the biospecimens to their sources. After the completion of the study, the specimens were moved back to the gross anatomical laboratory to be cremated. Human biospecimens were collected, stored, used, shared, and disposed of according to the informed consent signed by the subject or under a waiver of informed consent granted by the independent ethical review body, the institutional review board, or the ethics committee in accordance with 45 CFR 46–Protection of Human Subjects.

## 3 Results

### 3.1 Prototyping

We designed two prototypes incorporating the world’s smallest commercially available camera. The first design involved CI electrode arrays (J1–HiRes design) from Advanced Bionic. The optical system was integrated into the electrodes’ tips (Figures 1A, B). The extended portion of the array is embedded with biocompatible silicone material to ensure compatibility and integration, and it matches the nanocamera packaging. The second design is a slim probe with the nanocamera at its tip (Figure 1C). This prototype mimics a CI array without its contacts. It is designed to “probe” the cochlea before CI implantation by acquiring real-time video footage of the human cochlea during its insertion.

### 3.2 Performance of the nanocamera

**FIGURE 2:**
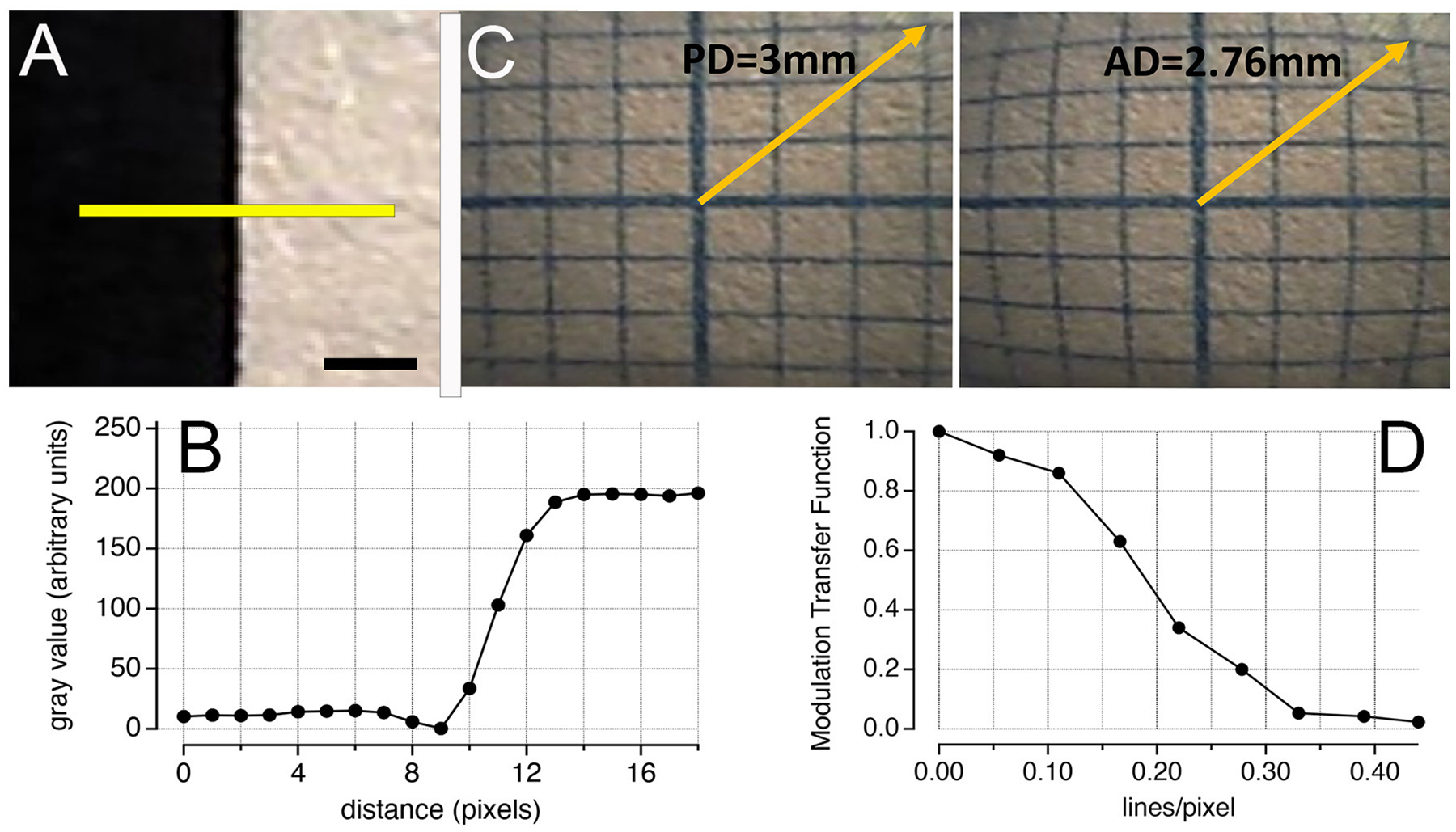
Performance of the nanocamera. **(A)** Image of a black–white transition. The yellow line was used to analyze the pixel profile using the software ImageJ. Scale bar: 0.5 mm. **(B)** Shows the pixel values along the transition in **(A)**. Using the edge response method, the spatial resolution was approximately 3 pixels. **(C)** Modulation transfer function obtained from **(B)**. **(D)** Barrel distortion of an image acquired at a 2-mm distance from the 1-mm graph paper. Comparing the distortion with the same image distortion-free (corrected by software), the effect was quantified at ~8.5%. PD, predictive distance; AD, actual distance.

The spatial resolution, determined with the edge response method from a color step of black to white, was ~5.25 μm, corresponding to ~3 pixels (Figures 2A, B). From this transition, we determined the MTF, illustrated in Figure 2D. The MTF evaluates how effectively an optical system can transfer detail and contrast from the scene to the image across different spatial frequencies. An MTF value close to 1 indicates that the system almost perfectly preserves the contrast and detail for the specific spatial frequency, reflecting an ideal or nearly ideal performance in transferring the original scene’s detail and contrast to the image. As can be seen in the graph, lines per pixel (lp/pp) quantifies the spatial frequency in terms of the number of distinguishable line pairs (one black and one white line) per pixel. For example, a value of 0.15 lp/pp implies that each line pair spans ~6.67 pixels, offering insight into the system’s resolution capabilities. At this spatial frequency, the MTF value is 0.7, indicating that the system preserves 70% of the contrast and detail from the original scene.

When analyzing the image of the 1-mm graph paper, we identified barrel distortion (Figure 2D). By quantifying the displacement of points from their expected positions in the distortion-free image, we determined that the effect of the distortion is ~8.5%.

**FIGURE 3:**
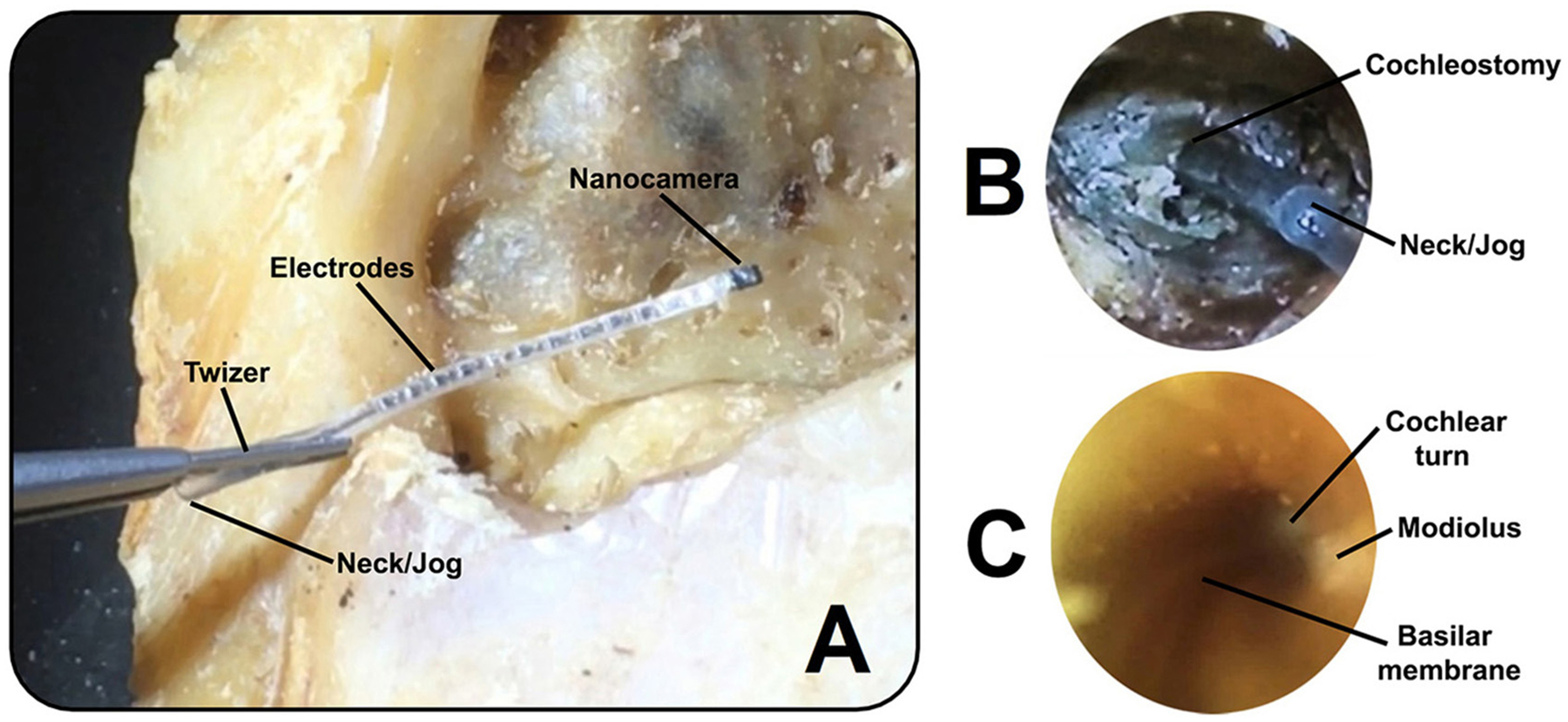
**(A)** Cochlear implant electrode array (first prototype) with the nanocamera before insertion into the human cochlea. **(B)** Full insertion of the modified cochlear implant electrode shown in **(A)** into the scala tympani, achieving an insertion depth of approximately 22 mm. **(C)** Image captured within the cochlea with the cochlear probe (second prototype) during insertion. The basilar membrane can be seen as a snail line along the conduit, with views of the modiolus and cochlear turn.

We fully inserted the CI electrode arrays with the nanocamera (first prototype; Figure 3A) in eight cadaveric human temporal bones (Figure 3B). Utilizing the second design, we captured real-time video footage during the probe’s insertion into the scala tympani of human cadaveric ears. Critical structures that can be seen include the basilar membrane, the modiolus, and the cochlear wall (Figure 3C). Results showed no malformations of the cochlea in the specimens analyzed.

**FIGURE 4:**
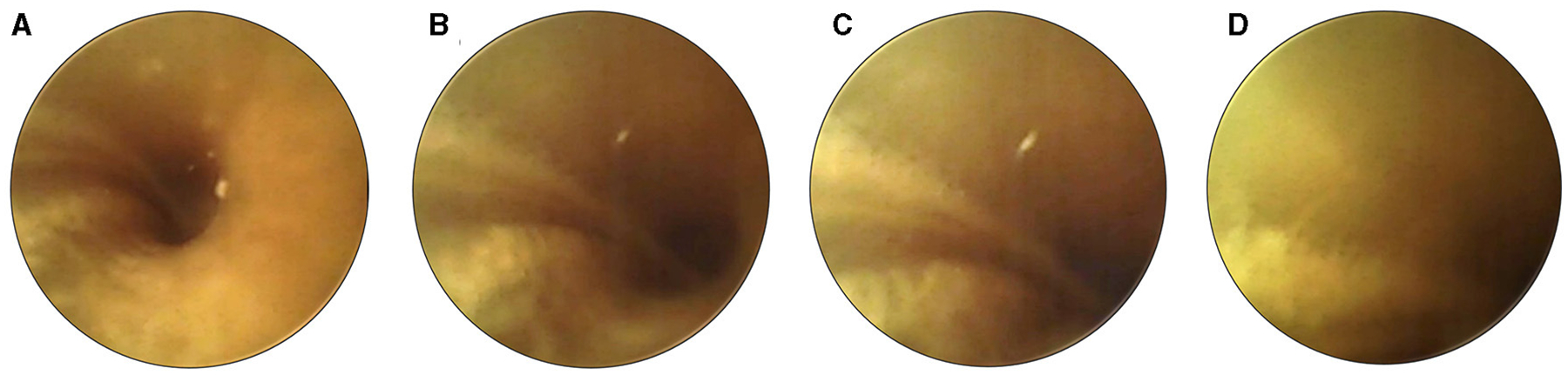
Sequential images illustrate the careful maneuvering of the cochlear probe (second prototype) during insertion. As the device approaches proximity with the cochlear wall **(A–D)**, the surgeon halts the insertion (as seen in image **D**). This pause allows necessary adjustments to the probe’s position to prevent or minimize potential damage.

The real-time video footage enabled the surgeon to navigate the slim probe in the cochlear turn, reducing its interaction with the delicate inner ear structures (Figures 4A–D). The surgeon could immediately stop inserting the probe when its tip approached the cochlear wall, modiolus, or any other sensitive structure (Figure 4D).

### 3.3 μCT imaging

**FIGURE 5:**
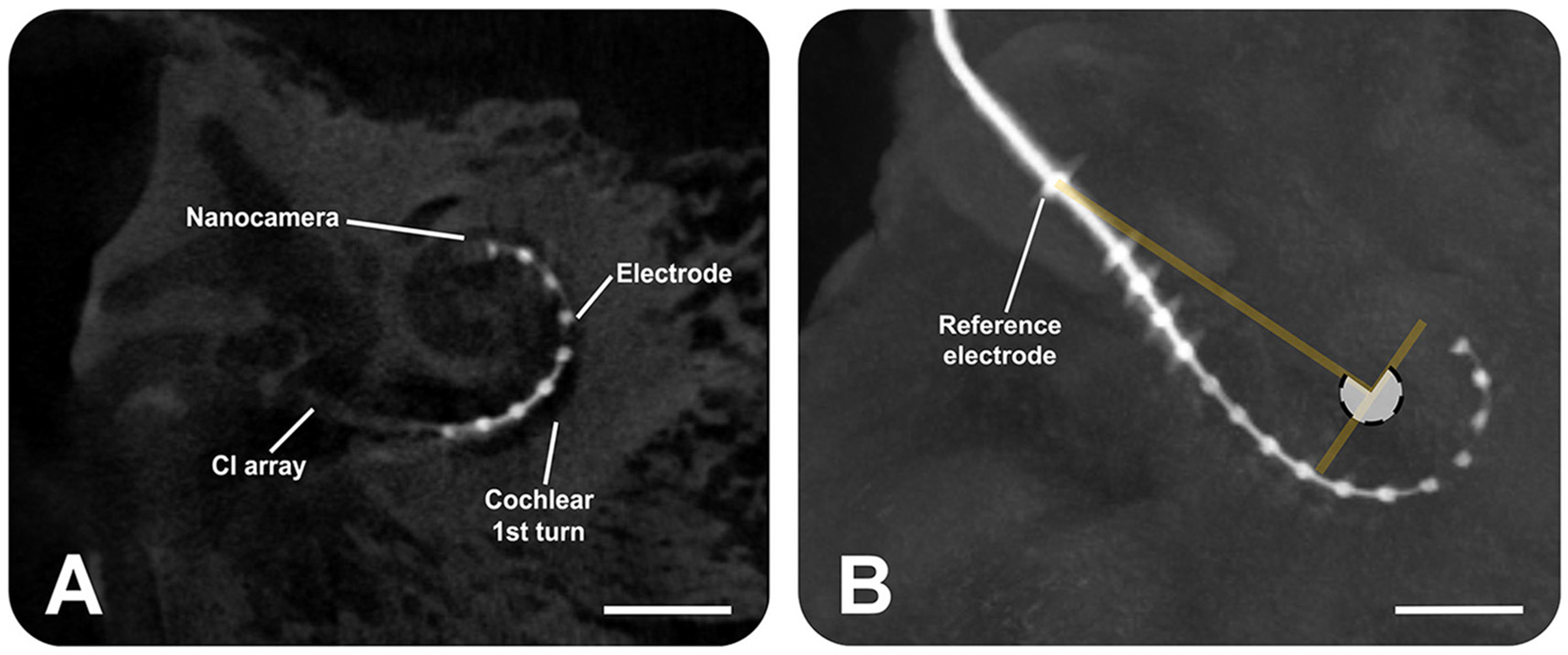
Micro-computed tomography scans show the insertion of the cochlear implant electrode array (Advanced Bionics) equipped with the nanocamera into the human cochlea. **(A)** Sagittal slice, providing a cross-sectional view. **(B)** Sagittal view from the three-dimensional volume rendering of the human temporal bone, highlighting the electrode. The angular insertion depth is ~270° as the white transparent circle shows. The scale bars represent 4 mm.

After the CI electrode array, equipped with the nanocamera, was inserted in the cochlea, μCT scans confirmed its positioning after insertion (Figure 5). The angular insertion depth is approximately 270° (Figure 5B).

## 4 Discussion

This article introduces a novel optical system that can be integrated into a CI electrode array to monitor its insertion process. The aim is to minimize surgical damage to the ear and prevent further hearing loss, which could adversely affect the performance of CI users. Benchtop evaluations confirm that integrating a nanocamera into conventional CI electrodes and cochlear probes does not compromise their compliance during insertion. This outcome is attributed to the selection of wire diameters used to connect the nanocamera, which falls within the range of those found in conventional CI array electrodes. Video recordings of the cochlear structures visible in scala tympani were acquired, and no scala migration was detected. Full insertions of the CI electrode with the nanocamera into cadaveric human temporal bones were successfully conducted and verified by high-resolution μCT scans.

The nanocamera implemented in our study does not have as many pixels as other miniaturized cameras on the market with a higher pixel count; however, that this optical system is the only one that fits into the cochlea and beyond the first turn is important to highlight. Given the cochlea’s limited size, the 200 × 200 pixels employed on a small field of view enable detailed visualization of the inner ear’s structures, as shown in Figures 3C, 4. Considering the performance of the nanocamera, the spatial resolution, determined using the edge response method, was ~5 μm, which translates to ~3 pixels. The minimum distance between two objects, at which the nanocamera can distinguish them as separate entities, is ~5 μm. When we translate this spatial resolution into the context of the MTF plot (Figure 2C), at a spatial frequency of 0.32 lines per pixel (lp/pp), we are looking at a scenario in which a white-and-black line pattern spans a 3-pixel distance. At this spatial frequency, the MTF significantly decreases to approximately 0.05. This reduction in MTF at 0.32 lp/pp—a frequency corresponding to the maximum spatial resolution—highlights a critical aspect of optical system performance: as we approach the limit of what the system can resolve, the contrast between closely spaced details is substantially compromised.

Regarding the optical effect from Figure 2C, we found a barrel distortion, characterized by image magnification decreasing with distance from the optical axis, causing objects to appear bowed outward toward the edges of the image. This phenomenon typically occurs when the magnifying power of the lens decreases too quickly with distance from its center. In the context of our nanocamera, this distortion is a consequence of the miniaturized lens design. Although this optical effect can introduce alterations, postprocessing the image further can improve its visualization.

With the imaging system, surgeons can now follow the CI electrode’s path during its insertion and reduce cochlear damage through early interventions and steering of the CI electrode. This technology not only aids in precise placement during surgery but also improves presurgical planning by offering a detailed view of the cochlea’s internal structure. Furthermore, it allows the electrode’s final depth and positioning to be carefully evaluated. It is critical to assess the potential for electrode migration over the implant’s lifespan and ensure optimal orientation toward the spiral ganglion neurons. At the recent Conference on Implantable Auditory Prostheses in California, the benefits of robotic CI electrode insertion over manual insertion have been discussed. Robotic insertion holds the promise of inflicting significantly less damage to the cochlea. However, a salient challenge for these robotic systems centers on guiding the insertion speed and providing feedback on the insertion process. Historically, anchored in force measurements, feedback could experience a paradigm shift with visual feedback through advanced camera systems, providing surgeons or the robot with a more intuitive and responsive insertion process.

Integrating micro-LEDs with the nanocamera at the device’s tip presents specific challenges regarding illumination. Initially, that the nanocamera alone, without any micro-LED attached, measures 0.65 × 0.65 mm is important to note. When the micro-LED from Evemodel is incorporated alongside the CI array and cochlear probe, the resultant thickness at the device’s tip increases to ~1.1 mm. In contrast, the smaller nano-point micro-LED reduces the thickness to ~0.85 mm compared to the larger light source model. This reduction is crucial, considering the cochlea’s confined space and the fact that any increase in size at the nanocamera’s location can significantly impact the ease of insertion, particularly around the cochlea’s first turn, where space is most restricted. A clear distinction emerges when examining the tip sizes of our modified CI arrays and cochlear probes against those of standard CI electrodes. Standard CI arrays have a smaller thickness at their tip (Shin et al., 2021). For example, Med-El (Austria) features a tip size of 0.4 × 0.5 mm^2^, Cochlear (Australia) at 0.3 mm, Todoc (South Korea) at 0.35 × 0.45 mm^2^, and Advanced Bionics (same electrode used) at 0.4 mm. Compared to these, our prototypes present a larger tip profile due to the nanocamera size and micro-LED, even after successful full insertions (Figure 3B), confirmed with μCT scans (Figure 5), and no scala migration observed in video acquisitions. This highlights the need for further progress in our designs.

Addressing the challenges presented by integrating micro-LEDs with the nanocamera, waveguides offer a valuable alternative for illumination in the cochlea. These waveguides can reduce the size-related issues associated with micro-LEDs, providing a more compact light delivery system. The success of these waveguides depends on their mechanical and optical properties, such as propagation and bending losses, which are crucial for effective performance in the cochlea’s confined space (Kampasi et al., 2021). Developing waveguides that meet these requirements is vital to enhance the design for easier insertion and improved functionality within the cochlea. The polymer OrmoComp (Kayaku Advanced Materials, Westborough, USA) emerged as a promising solution. This polymer is an ormocer, a material that merges the qualities of organic polymers with inorganic ceramics. Upon UV curing, it exhibits properties similar to glass, reflecting the durability and stability of ceramics yet maintaining the flexibility of organic materials. This combination results in a medium that is both thermally and chemically stable and optically highly transparent and is suitable for light transmission across visible and near-infrared wavelengths (Heinrich, 2021). Furthermore, its biocompatibility makes it a promising material for medical applications (Schizas and Karalekas, 2011).

Given these promising attributes, we are currently developing and characterizing waveguides with OrmoComp as the core, featuring a 100-μm inner diameter and a 16-μm thickness polyimide cladding. Alternative cladding materials, such as the fluoropolymer CYTOP (AGC Inc. Chemicals Company, Tokyo, Japan) and UV-curable resins, are also being explored (Evertz et al., 2021). As our research progresses, we are focusing on critical aspects, such as the waveguides’ resistance to photobleaching and their stability in electrolytic environments resembling cochlear perilymph, and evaluating their long-term performance and biocompatibility through studies in animal models. These investigations are crucial in comprehensively determining the suitability of waveguides for clinical applications in which a miniaturized means of light delivery is required, as in our study.

While the current design of our optical system has enabled successful full insertions into human temporal bones, there is still potential for further improvements. A recent advancement comes from Omnivision, which released a new nanocamera design called OCHT10. This model maintains the same package size as the OVM6948 but doubles the pixel count, increasing the resolution to 400 × 400. This enhancement in resolution is attributed to a smaller manufacturing process that allows for a pixel size reduction to approximately 1 μm. Furthermore, the new design achieves a 20% reduction in power consumption, lowering it to approximately 20 mW, which marks a considerable improvement over the previous model. This progress in quality image by increasing the spatial resolution (smaller pixel size) can help capture finer details. Such an enhancement has the potential to improve the MTF of the optical system, allowing for a more precise depiction of details at higher spatial frequencies. Nonetheless, that MTF performance is not solely determined by pixel resolution is important to highlight. The overall quality of the optical system plays a crucial role, including factors such as the lens design, diffraction limit, and inherent sensor noise (Simon, 2019).

Another potential enhancement under exploration is the reduction of the nanocamera’s dimensions, targeting a form factor smaller than a CI electrode’s ring contact. This strategy for miniaturization could be feasible by adopting the 1-μm pixel size utilized in the OCHT10 while maintaining a resolution of 200 × 200 pixels, similar to that of the OVM6948. Reaching such a compact design could significantly benefit surgical procedures by reducing contact with the basilar membrane at critical turns and enhancing the ease of maneuvering the device during surgery.

Continuing this trend of miniaturization, achieving an even smaller packaging nanocamera size combined with increased resolution might involve further reducing the pixel dimension. Recent advancements in micro-manufacturing processes have made ultra-small pixel sizes possible, as seen in Samsung’s development of the world’s smallest pixel (0.56 μm). However, pixel size reduction is not without its set of challenges. Smaller pixel sizes can lead to several constraints, each impacting image quality. First, increased shot noise, resulting from the quantum nature of photons, is more pronounced in smaller pixels, causing grainier images (Chen et al., 2000). Second, the smaller surface area of each pixel leads to diminished light sensitivity, affecting the image sensor’s dynamic range. This reduction in dynamic range limits the sensor’s ability to capture a broad spectrum of light intensities, which is crucial for detailing the brightest and darkest areas of visual information (Chen et al., 2000). Third, optical crosstalk becomes a significant concern as pixel proximity increases. This inconvenience arises when light intended for one pixel inadvertently influences adjacent ones, especially at steep angles, and reduces image contrast (Hirakawa, 2008). Fourth, the quantum efficiency, or the ability of the sensor to convert light into an electronic signal, decreases as light hits the sensor at steeper angles. This effect is more pronounced in smaller pixels, leading to inconsistent image quality across different sensor areas (Bianconi et al., 2022). In addition to these pixel-related concerns, making the optical system smaller requires carefully designing lenses to avoid optical aberrations, such as distortion, that can compromise the image quality. Addressing these complex challenges is essential in image sensor design to ensure that the benefits of miniaturization do not come at the cost of reduced imaging capabilities in medical applications.

Further enhancements to the optical system are on the horizon, expanding its potential beyond insertion monitoring. One promising path of exploration is the evaluation of cochlear health through the phenomenon known as birefringence. *Birefringence* refers to the differential refraction of light as it passes through anisotropic materials, resulting in a change in the polarization state of the incident light. In the context of the cochlea, the basilar membrane exhibits birefringence due to the organized orientation of its collagen fibrils (Kalwani et al., 2013). The unique patterns of light interference, as they interact with this biological structure, can provide critical insights into the health and integrity of the cochlea. Remarkably, no diagnostic optical probe is specifically tailored for this purpose within the cochlea. The development and integration of such an optical system would not only be groundbreaking, but it could also serve as a tool for clinicians to evaluate cochlear health. This assessment would provide vital information about the basilar membrane’s condition before and after implantation, allowing for the anticipation and prevention of postoperative complications in CI users.

In conclusion, integrating optical systems into CI electrodes significantly advances auditory prosthetics. This novel approach, offering real-time visual feedback during the implantation process, can enhance surgical precision and potentially reduce damage to the cochlea. While it is anticipated to contribute to preserving residual hearing, clinical studies are essential to understand its impact fully. Beyond the immediate surgical applications, these optical systems also promise to advance cochlear health assessment by providing valuable insights for postoperative care. Moreover, preserving the integrity of the inner ear structures benefits CI users and opens doors to future therapeutic strategies, such as gene delivery into the inner ear (Kanzaki, 2018; Lahlou et al., 2023). This development, at the forefront of CI technology, could improve surgical techniques and outcomes for CI users’ experience, marking an important step toward optimizing auditory prosthetics.

## Data Availability

The original contributions presented in the study are included in the article/supplementary material, further inquiries can be directed to the corresponding author.
